# In Vivo Knockdown of TAK1 Accelerates Bone Marrow Proliferation/Differentiation and Induces Systemic Inflammation

**DOI:** 10.1371/journal.pone.0057348

**Published:** 2013-03-07

**Authors:** Paul M. Vink, Wendy M. Smout, Lilian J. Driessen-Engels, Alex M. de Bruin, Dianne Delsing, Magda A. Krajnc-Franken, Aswin J. Jansen, Eric F. Rovers, André A. van Puijenbroek, Allard Kaptein, Martijn A. Nolte, Anja Garritsen, Hans van Eenennaam

**Affiliations:** 1 Department of Immune Therapeutics, Merck Research Laboratories, MSD, Oss, The Netherlands; 2 Department of Experimental Immunology, Academic Medical Center, Amsterdam, The Netherlands; 3 Department of Toxicology and Drug Disposition, Merck Research Laboratories, MSD, Oss, The Netherlands; 4 Department of Molecular Pharmacology and DMPK, Merck Research Laboratories, MSD, Oss, The Netherlands; 5 Department of Hematopoiesis, Sanquin Research and Landsteiner Laboratory, Amsterdam, The Netherlands; University of Kansas Medical Center, United States of America

## Abstract

TAK1 (TGF-β Activated Kinase 1) is a MAPK kinase kinase, which activates the p38- and JNK-MAPK and NF-κB pathways downstream of receptors such as Toll-Like-, cytokine- and T-cell and B-cell receptors. Representing such an important node in the pro-inflammatory signal-transduction network, the function of TAK1 has been studied extensively. TAK1 knock-out mice are embryonic lethal, while conditional knock-out mice demonstrated either a pro- or anti-inflammatory function. To study the function of TAK1 protein in the adult immune system, we generated and characterized a transgenic mouse expressing TAK1 shRNA under the control of a doxycycline-inducible promoter. Following treatment of TAK-1 shRNA transgenic mice with doxycycline an effective knockdown of TAK1 protein levels was observed in lymphoid organs and cells in the peritoneal cavity (>50% down regulation). TAK1 knockdown resulted in significant changes in leukocyte populations in blood, bone marrow, spleen and peritoneal cavity. Upon TAK1 knockdown mice demonstrated splenomegaly, signs of systemic inflammation (increased levels of circulating cytokines and increase in cellularity of the B-cell areas and in germinal center development in the follicles) and degenerative changes in heart, kidneys and liver. Not surprisingly, TAK1-Tg mice treated with LPS or anti-CD3 antibodies showed enhanced cytokine/chemokine secretion. Finally, analysis of progenitor cells in the bone marrow upon doxycycline treatment showed increased proliferation and differentiation of myeloid progenitor cells. Given the similarity of the phenotype with TGF-β genetic models, our data suggest that in our model the function of TAK1 in TGF-β signal-transduction is overruling its function in pro-inflammatory signaling.

## Introduction

Mitogen-Activated Protein Kinases (MAPKs) and Nuclear Factor-κB (NF-κB) orchestrate cellular responses of the host immune system to environmental changes. The MAPKs and the NF-κB pathways can be activated by stress, bacterial infection, viral infection and by cytokines, thereby mediating cellular responses, such as proliferation, differentiation, survival and transcription of pro-inflammatory mediators [1]–[5].

TAK1 (TGF-β Activated Kinase) is a MAP3K, which can activate the p38- and JNK-MAPKs and NF-κB pathways [6]–[7]. TAK1 is activated downstream of various receptors, such as the TGF-β receptor, Toll-Like receptors, IL-1 receptor, NOD2, TNF receptor, T-cell receptor and B-cell receptor [8]–[10]. Upon binding by their respective ligands, these receptors recruit TRAF proteins, which activate TAK1 by polyubiquitination [11]. Once activated TAK1 phoshorylates IκB kinases, which in their turn phosphorylate IκB protein, resulting in the degradation of these proteins. As a result of the degradation of the IκB proteins, NF-κB translocates from the cytoplasm to the nucleus to drive gene transcription [7], [12]. In parallel, active TAK1 phosphorylates MAPK kinases MKK3/6 and MKK4/7, which subsequently phosphorylate p38 and JNK MAPKs [7], [13]. Activated p38 and JNK kinases induce gene transcription by phosphorylation of nuclear transcription factors, such as Myc, ATF-2 and c-Jun [4]–[5].

Acting upstream of these crucial signal-transduction pathways, TAK1 has been the subject of many studies in vitro and in vivo. However, a full understanding of TAK1 function is hampered by the observation that genetic disruption of TAK1 in mice results in an embryonic lethal phenotype [14]–[15]. Subsequent studies using cell-type specific conditional knock-out mice revealed that TAK1 regulates skin inflammation and keratinocyte death [16]–[17], is critical for the generation of thymic-derived regulatory T-cells [18] and the maturation of single-positive CD4+ and CD8+ thymocytes [19], mediates T-cell receptor dependent proliferation and cytokine dependent p38 activation [20], is essential for MDP-induced NOD2 activation [21], prevents epithelium apoptosis and the development of ileitis and colitis [22]–[23], is involved in fibrogenic responses [24], is protective against chemical-induced colitis [25], is essential for B-cell maturation and activation [26], protects for liver injury, inflammation and fibrosis [27] and is involved in hair follicle morphogenesis and hair growth [28]. In addition, using an inducible knock-out TAK1 deletion was shown to induce bone marrow and liver failures due to massive apoptotic cell-death of the bone marrow and hepatocytes [29]. Recently, a novel function for TAK1 was described in Th1 and Th17 differentiation upon targeting of TAK1 in monocyte specifically [30].

In human pathology, targeting of TAK1 is often mentioned in the context of autoimmune disease. Extensive in vitro characterization demonstrated a crucial role for TAK1 in pro-inflammatory signaling, such as induced by TLRs, NOD2, IL-1β and TNF-α receptors, T-cell and B-cell receptors [8]–[10]. In addition, TAK1 has been implicated in the activation of rheumatoid arthritis synoviocytes and human articular cartilage [31]–[32]. Recently, with the limited efficacy of p38α inhibitors in Phase 2 clinical studies in patients suffering from rheumatoid arthritis, suggestions were made to target kinases higher in the pathways, e.g. TAK1 [33]. The data collected using cell-type specific TAK1 genetic disruption suggest that TAK1 has both a pro- and anti-inflammatory function. To address the question whether systemic TAK1 inhibition in an adult immune system will demonstrate a pro- or anti-inflammatory phenotype, we generated and characterized a transgenic mouse expressing a TAK1 shRNA under control of a doxycycline-inducible reporter.

## Materials and Methods

### Doxycycline-inducible TAK1 shRNA transgenic mice

Transgenic mice expressing TAK1 shRNA under control of a doxycycline inducible promoter were generated as described in detail by Seibler and coworkers at Taconic Artemis in Cologne, Germany [34]–[35]. In short, an exchange vector was generated that has the following order in 5′ to 3′ direction: synthetic poly(A) signal, F3-site, neomycin-resistance gene lacking the start ATG, hGH polyadenylation signal, H1 promoter with a tet-operator sequence, TAK1-specfic shRNA sequence (5′-AGATGTCGCTATTAAACAGATTCAAGAGATCTGTTTAATAGCGATC-3′), five thymidines, loxP-site, codon optimized tet-repressor gene under control of CAGGS promoter, synthetic poly(A) signal, loxP-site and FRT-site. Next, the exchange vector was transfected into ES cells to accomplish recombinase-mediated cassette exchange in the *rosa26* locus. ES cells were selected that carry shRNA under control of the H1-tet promoter, the codon optimized tet-repressor gene under control of the CAGGS promoter and a truncated neomycin resistance gene for selection upon successful RMCE. Transfection and injection protocols were applied as described previously [34]–[35]. Generated mice were on a B6/129S2F1 mixed background. Mice were housed in the animal facility of MSD, Oss, the Netherlands in open cages with standard food and drinking water ad libitum. All experiments were approved by the Animal Welfare Committee of MSD, Oss, the Netherlands.

Induction of TAK1 shRNA expression was performed by addition of 0.2 mg/ml doxycycline hydrochoride (Sigma-Aldrich) to the drinking water supplemented with 10% sucrose for two or five weeks.

### Inflammatory mouse models

Acute inflammation was induced by injection of mice intraperitoneal (i.p.) 1 mg/kg LPS (Sigma L-2880) in PBS. After 1.5 hours, mice were anesthetized (0.2 l/min O_2_, 0.4 l/min medical air +2.5% isoflurane) and blood was sampled by eye extraction. After blood sampling mice were sacrificed by cervical dislocation and organs were harvested.

In a second model acute inflammation was induced by injection of mice with 5 µg purified hamster anti-mouse CD3 monoclonal antibody (clone 145-2C11, no azide/low endotoxin art.nr. 553057, BD Biosciences). After 3 hours, mice were anesthesized and blood was sampled by eye extraction. After blood sampling mice were sacrificed by cervical dislocation organs were harvested.

### Isolation and characterization of mouse tissues

Cells were isolated from harvested spleens by dissociation using PBS/0.5% BSA and subsequent centrifugation at 1,000 rpm for 10 min. Splenocytes were resuspended in 1 ml 0.16 M NH_4_CL and 0.01 M KHCO_3_ and incubated for 3–5 min on ice to lyse erythrocytes, followed by neutralization in 10 ml PBS/0.5% BSA. After centrifugation, splenocytes were resuspended in PBS/0.5% BSA for analysis by flow cytometry or washed one time with ice-cold PBS and lysed in Cell Extraction buffer (FNN0011, Biosource) supplemented with 1 mM PMSF and Protein Kinase inhibitors (Complete EDTA free, Roche). After 30 min lysates were centrifuged at 12,000 g for 10 minutes at 4°C and supernatants were transferred to a clean tube. Similar protocols were followed to isolate cells from lymph nodes and thymus, except now without erythrocyte lysis step. Peritoneal cells were harvested by injection of 3.5 ml cold buffer (PBS +0.5% BSA) in the abdomen, followed by gently massaging. Peritoneal cells were isolated by aspriraton of peritoneal fluid using a 5 ml syringe and 23G needle.

Isolated blood was characterized by flow cytometry. 50 µL of isolated blood was plated out in a v-well bottom plate and red blood cells were lysed using 150 µL ice-cold red blood cell lysis buffer for 5 minutes at 4°C. Cells were centrifuged at 1,200 rpm for 3 minutes at 4°C and subsequently resuspended in 200 µL red blood cell lysis buffer for 5 minutes at 4°C. Cells were centrifuged 1,200 rpm for 3 minutes 4°C and stained like described below for splenocytes.

Harvested livers and skin were lysed by means of the Mini Bead Beater in Cell Extraction buffer (FNN0011, Biosource) supplemented with 1 mM PMSF and Protein Kinase inhibitors (Complete EDTA free, Roche). After 30 min lysates were centrifuged at 12,000 rpm for 10 minutes at 4°C and supernatants were transferred to a clean tube.

Total protein amounts in the isolated lysates were determined using BCA protein assay (Pierce) following the manufacturer's instructions. Protein lysates (20 µg) were mixed 3∶1 with Laemmli buffer (BioRad) supplemented with 200 mM DTT, followed by heating for 5 minutes at 95°C. Proteins were separated on a 4–12% NuPage gel (Invitrogen) in MOPS running buffer and transferred to PVDF membrane (Millipore) for 1 hour using a mini Transblot electrophoretic transfer cell, at 100 Volt in blotbuffer (3.02 g/L Tris, 14.4 g/L Glycine, 10% Methanol).

For TAK1 detection, membranes were blocked for 30 minutes at room temperature with 5% skim-milk (Difco) in TBS-Tween (10 mM Tris-HCl, 0.1 M NaCl, 0.1% Tween-20, pH 7.4). To detect TAK1, TAK1 antibody (Santa Cruz SC-7162; 1∶1,000 dilution in blocking buffer) was added to the membrane and incubated overnight at 4°C. To monitor loading efficiencies, western blots were probed in parallel for β-actin (Cell Signaling, #49671). Next, the blot was washed three times with ECL wash buffer (10 mM Tris-HCl, 0.1 M NaCl, 0.5% Tween-20, pH 7.4) and incubated with detection antibody (goat anti-rabbit Ig HRP conjugate, Biosource ALI 4404; 1∶20,000 dilution in blocking buffer) for 1 hour at room temperature. Again, blots were washed three times 5 minutes with ECL washbuffer and bound immunoglobulins were visualized using ECL reagent (Pierce).

The composition of isolated cells was studied using flow cytometry. Single cell solutions were made in PBS/0.5% BSA (2.10^6^ cells/ml). 25 ng/100,000 cells Fc-block was added (cat no 553142) to block Fc receptors. Subsequently, cells were labeled for 30 min at 4°C in PBS/0.5%BSA in the dark using antibodies specifically recognizing CD3 (BD-553061, BD-552774), CD19 (BD-553786), CD4 (BD-557308, BD-552051), CD8 (BD-553031, BD-553030), CD11b (BD-557397), F4/80 (Serotec, MCA497F), Ly6G (BD-551460, BD-551461), B220 (Southern, 1665-11), CD62L (BD-553151), CD44 (BD-553134), AA4.1 (e-Biosciences, 25-5892), CD23 (Southern, 1585-09), IgM (BD-553437), CD5 (BD-550035) and MHCII (e-Biosciences, 11-5321-81). Next, cells were centrifuged and resuspended in PBS/0.5% BSA containing Cell Viability solution (7-amino-actinomycin (7-AAD, BD-555815). Fluorescence was detected using a FACSCanto II cytometer (Beckton Dickinson). The intensity of minimally 10,000 cells was measured. Dead cells were eliminated from the analysis. Data analysis was performed using FACSDiVa 5.1 software (Beckton Dickinson).

### TAK1 mRNA detection

Total RNA from liver samples (±10 mg), splenocytes and thymocytes (1.10^7^ cells) were isolated using the Trizol method. cDNA was generated by mixing 1 µg of RNA, 0.5 µg/ml Oligo dT (Promega) and 0.5 µg/ml random hexamers (GE Healthcare Biosystems). Samples were heated for 10 minutes at 70°C and subsequently cooled on ice. Next, M-MLV Reverse Transcriptase, RNase H Minus, Point Mutant (Promega) and 10 mM dNTP set ultrapure (GE Healthcare Biosystems) was added and the total mixture was incubated for 10 minutes at 25°C, followed by 1 hour at 42°C and 10 minutes at 70°C.

To quantitate TAK1 mRNA levels, cDNA was diluted to 2 ng/ml cDNA or 10 ng/ml cDNA for standard curve (√10 dilutions, per tissue pooled samples from wildtype animals). The PCR mastermix was prepared using 12.5 µl Sybrgreen mix, 3 µM (2.5 µl) forward primer (5′-CTC ATG CCA TGA GCT GGT GT-3′), 3 µM (2.5 µl) reverse primer (5′-GGT TTC CGG CGT GTT ATC ACT-3′) and 2.5 µl milli Q. To detect HP1 mRNA, for normalization the following forward primer (5′-GCC CAA GAT GGA CGC AAT C-3′) and reverse primer (5′-CCG AGG CGC CAG TCT TC-3′) were used. In a 96-well plate 20 µl PCR mix and 5 µl standard or sample (2 ng/µl) was added and amplified using ABI Prism 7900HT. The following cycles were run to amplify the TAK1 cDNA: 10 min at 95°C, 40 cycles of 15 sec at 95°C, 1 min at 60°C, followed by dissociation step of 15 sec at 95°C, 15 sec at 60°C and 15 sec at 95°C.

### Cytokine detection

Mouse cytokines were detected using the mouse Bio-Plex Mouse Cytokine 23-plex Panel (#M60009RDPD) according to the manufacturer's instructions.

### In vivo Br-dUTP labeling

To study the proliferation and differentiation of bone marrow cells, BrdU (Sigma, 858811-5G) was injected i.p. (0.17 mg/g bodyweight) in 200 µl PBS. Mice (n = 5 mice) were sacrificed after 24 hours and blood, spleen, and bone marrow was isolated. Bone marrow was isolated in PBS supplemented with 0,5% BSA by crushing bones with a mortar and pestle followed by filtration of the suspension over a 70 µm filter. For analysis of progenitor cells, bone marrow cells were stained using the following antibodies: c-Kit (2B8), Sca-1 (D7), CD34 (RAM34), CD16/32 (93), Flt3 (A2F10), CD4 (GK1.5), CD8α (53-6.7), B220 (RA3-6B2), CD11b (M1/70), Gr1 (RB6-8C5), and Ter119 (Ly-76). All antibodies were obtained from eBioscience. For BrdU detection, cells were subsequently fixed and permeabilized with the Foxp3 Fixation/Permeabilization set (eBioscience), treated with 50 kU DNAse for 10 minutes at room temperature and 30 minutes on ice, washed and stained with anti-BrdU for 30 minutes at room temperature (3D4, BD Biosciences). Flow cytometry analyses were performed on FACSCanto II (BD Biosciences) and data were analyzed using FlowJo software (Tree Star).

Cells isolated from spleen and blood (see above) were labeled using antibodies specifically recognizing CD4 (BD-552051), CD8 (BD-553033), Ly6G (BD-551461), B220 (BD-552094, BD-552772), AA4.1 (e-Biosciences, 17-5892-83), CD23 (Southern, 1585-09), IgM (BD-552867), CD5 (BD-550035), 7/4 (Serotec-MCA771A647) and CD138 (BD-558626). Next, cells were washed with PBS/0.5%BSA, centrifuged and fixed with 70% EtOH in PBS for 30 min at 4°C. Again cells were washed with PBS/0.5%BSA, centrifuged and permeabilized with 1% paraformaldehyde and 0.01% Tween 20 in PBS overnight at 4°C. Next, cells were washed with PBS/0.5%BSA, centrifuged and resuspended in DNAse solution (0.15 M NaCl (Acros organics 207790010), 4.2 mM MgCl_2_ (Sigma M-8266), containing 50 kU DNAse (Roche-10104159001), incubated for 10 min at room temperature and 30 min at 4°C. Cells were washed, centrifuged and stained with anti-BrdU (eBioscience 11-6071) antibody and Fc-block (cat no 553142) for 30 min at RT. Fluorescence was detected using a FACSCanto II cytometer (Beckton Dickinson). The intensity of minimally 10,000 cells was measured. Data analysis was performed using FACSDiVa 5.1 software (Beckton Dickinson).

### Histopathology

Wild type and TAK1 transgenic animals were treated for five weeks with doxycycline in the drinking water to induce TAK1 knockdown. At autopsy, anaesthetized mice were sacrificed by exsanguination via the vena cava. The femur, heart, kidneys, liver, lungs, mesenteric lymph node, spleen, and thymus from all animals were dissected free of adjacent fat and other surrounding tissues and preserved in 4% buffered formaldehyde. The femur and sternum were put in a decalcification (Kristensen) solution for approximately 2 weeks. All samples were dehydrated and embedded in paraffin wax. Sections (3- to 4 µm-thick) made from these blocks were stained with hematoxylin and eosin (HE) staining method using standard procedures and examined histopathologically.

### Statistical significance

Statistical significance was established using the Mann-Whitney test. In cases where a pre-defined statistical hypothesis could be formed a two-tailed test was performed to determine statistical significance. Statistical test used for each experiment is indicated in the legend of the figures.

## Results

### In vivo TAK1 shRNA knockdown

**Figure 1:**
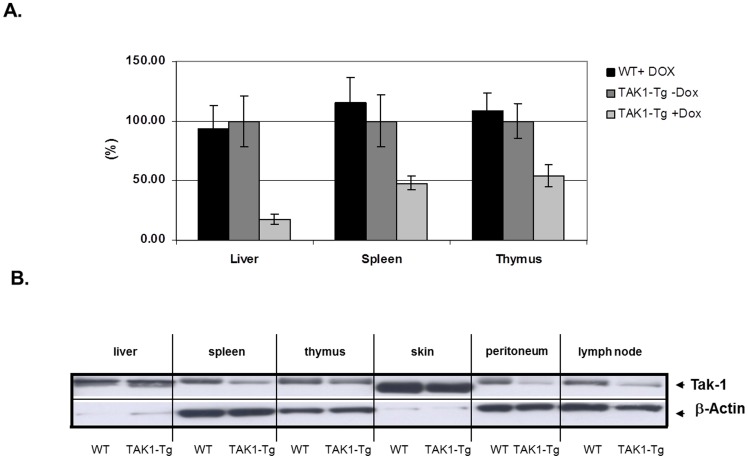
Induction of TAK1 knock-down in vivo. Transgenic mice expressing TAK1 shRNA under the control of a doxycycline-inducible promoter were generated. Expression of TAK1 shRNA was induced in adult mice by addition of 0.2 mg/ml doxycycline hydrochloride in the drinking water for two weeks. Mice were sacrificed and organs of interest were harvested. Knock-down of TAK1 was determined in wild type mice treated with doxycycline (WT +Dox), TAK1 shRNA transgenic mice treated with doxycycline (TAK1-Tg +Dox) or without doxycycline (TAK1-Tg –Dox). *A.* TAK1 mRNA levels were detected in liver, spleen and thymus by Q-PCR. The levels of HP1 mRNA was used for normalization. The average of the mRNA levels detected in samples derived from TAK1-Tg that were not treated with doxycycline were used as reference (100%). *B*. TAK1 protein levels were determined in liver, spleen, thymus, skin, lymph nodes and cells isolated from the peritoneal cavity using western blotting. β-actin levels were used to check for loading efficiency. Data are representative of at least three independent experiments.

To understand the role of TAK1 during inflammation in vivo, genetically engineered mice were generated expressing TAK1 shRNA under control of a doxycycline inducible promoter. Adult TAK1-Tg mice were treated with doxycycline for two weeks to induce expression of TAK1 shRNA. As shown in Figure 1A, TAK1 mRNA levels were reduced in liver, spleen and thymus of TAK1-Tg mice treated with doxycycline (TAK1-Tg +Dox) as compared to untreated TAK1-Tg mice (TAK1-Tg −Dox) or wild type mice treated with doxycycline (WT +Dox). As a consequence TAK1 protein levels were decreased in spleen and thymus, while TAK1 protein in these tissues was left unaffected in TAK1-Tg −Dox and WT +Dox mice (see Figure 1B). Similar reduction of TAK1 protein levels were observed in cells isolated from the peritoneal cavity and the lymph nodes. Only marginal reduction (30%) of TAK1 protein in the liver was found. Taken together, TAK1 protein levels were reduced with on average 50% in different immunological tissues after doxycycline treatment of TAK1 shRNA transgenic mouse.

### TAK1 knockdown dramatically changes immune cell subpopulations

To investigate whether knockdown of TAK1 affected the cellular composition of immune cells, blood, thymus, spleen, mesenteric lymph node and bone marrow were isolated from adult TAK1-Tg mice and WT mice, which were treated with doxycycline for five weeks.

In the thymus, no difference in the amount of CD3^+^ cells, CD4 SP and CD8 SP cells was observed when thymocytes from TAK1-Tg +Dox mice were compared with WT +Dox mice (data not shown).

In blood, a strong increase in the amount of neutrophils and monocytes was observed, which was accompanied by a reduction of B-cells and CD8^+^ T-cells (see Table 1). The amount of CD4^+^ T-cells was not changed as compared to WT mice. In addition, erythroid parameters in blood, such as hemoglobin concentration and number of reticulocytes, immature reticulocyte fraction, red blood cells and platelets were analyzed. No significant changes in these erythroid parameters were observed in TAK1-Tg vs. WT mice (data not shown).

**Table 1:** TAK1 knockdown affects the homeostasis of the immune system.

Tissue		Percentage of sub-population			Estimated mass of sub-population		
	celluar				Spleen^1^		
	subpopulation	(%)			(mg)		
		WT	TAK1-Tg	p-value	WT	TAK1-Tg	p-value
		(average)	(average)		(average)	(average)	
*blood*							
	neutrophils	10.34	35.72	<0.0001			
	monocytes	3.63	5.82	<0.0005			
	B-cells	64.94	42.18	<0.0001			
	CD4^+^	4.57	4.64	n.s.			
	CD8^+^	6.29	4.09	<0.02			
*spleen*							
	neutrophils	1.72	5.02	<0.0001	1.5	6.0	<0.0001
	monocytes	0.69	1.27	<0.0002	0.6	1.5	<0.0001
	B-cells	55.35	62.48	<0.004	48.0	74.7	<0.0001
	CD4^+^	22.54	17.89	<0.0002	20.5	21.0	n.s.
	CD8^+^	14.57	9.36	<0.0001	12.5	10.1	<0.009
*m-lymph node*							
	neutrophils	0	0.2	n.s.			
	monocytes	0.2	0.3	n.s.			
	B-cells	39.2	34.3	n.s.			
	CD4^+^	31.85	40.75	<0.001			
	CD8^+^	25.6	21.3	<0.03			
*bone marrow*							
	neutrophils	30.5	44.2	<0.01			
	monocytes	10.8	15.7	<0.01			
	B-cells	14.4	8.2	<0.001			
	CD5^+^	2.0	1.0	<0.001			
	CD4^+^	1.1	1.3	n.s.			
	CD8^+^	1.2	0.5	<0.001			

n.s. = not significant;

1 mass of subpopulation = % of sub-population x weight of spleen.

**Figure 2:**
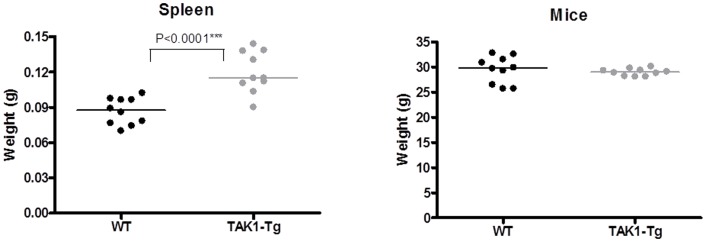
TAK1 knockdown affects cellular composition of different immunological compartments. Wildtype (WT) and TAK1 shRNA transgenic mice (TAK1-Tg) were treated with 0.2 mg/ml doxycycline in the drinking water for five weeks. Mice were sacrificed and organs harvested. Weights of the spleen and mice for WT and TAK1 mice is presented (n = 10 for each group). Statistical significance was determined using the Mann-Whitney test (p<0.05). Data are representative of at least two independent experiments.

Immediately apparent upon isolation of the spleens of TAK1-Tg mice was the increased size as compared to WT mice. As shown in Figure 2A, spleens of TAK1-Tg were ∼30% increased in weight, while no difference in body weight of WT and TAK1-Tg mice were found. Using flow cytometry more neutrophils, monocytes and B-cells were detected in the spleen (see Table 1). Similar amounts of CD4^+^ T-cells, while reduced numbers of CD8^+^ T-cells were observed in WT vs. TAK1-Tg mice. No difference was observed in the amount of splenic macrophages (data not shown).

As shown in Table 1, similar numbers of neutrophils, monocytes and B-cells were found in the mesenteric lymph node. At the same time, increased number of CD4^+^ T-cells and decreased number of CD8^+^ T-cells were found in the mesenteric lymph node. In the bone marrow increased numbers of monocytes and neutrophils, similar amounts of CD4^+^ T-cells, and reduced numbers of B-cells (CD19^+^ and CD5^+^) and CD8^+^ T-cells (see Table 1) were found. In conclusion, TAK1 knockdown dramatically affected the cellular subpopulations of the immune system, with increased numbers of neutrophils and monocytes and decreased numbers of CD8^+^ T-cells in blood, spleen and bone marrow and accumulation of B-cells in the spleen as most prominent phenotype.

### TAK1 knockdown induces systemic inflammation

**Figure 3:**
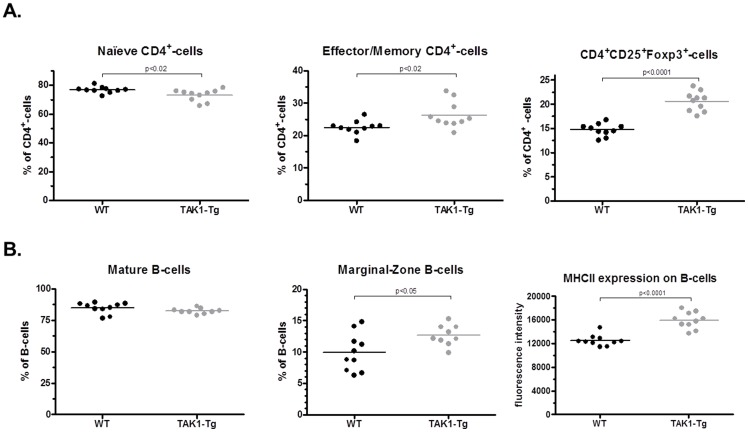
TAK1 knockdown shows more activated T- and B-cells in the spleen and bone marrow. Wildtype (WT) and TAK1 shRNA transgenic mice (TAK1-Tg) were treated with 0.2 mg/ml doxycycline in the drinking water for five weeks. Mice (n = 10 in each group) were sacrificed and spleens and bone marrow were harvested. Characterization of T- and B-cells was performed by flow cytometry. The percentage of naive T-cells (CD4^+^CD62L^+^CD44^int^), effector/memory T-cells (CD4^+^CD62L^−^CD44^+^) and regulatory T-cells (CD4^+^CD25^+^Foxp3^+^) are presented as % of total CD4^+^ cells in panel *A*. The percentage of mature B-cells (B220^+^AA4.1^−^CD23^+^IgM^low^), Marginal zone B-cells (B220^+^AA4.1^−^CD23^−^IgM^high^) and MHCII-expression on B220^+^ cells (in fluorescence intensity) are depicted in panel *B*. Statistical significance was determined using the Mann-Whitney test (p<0.05). Data are representative of at least two independent experiments.

Next, the activation status of cells present in the lymphoid tissues was determined. As shown in Figure 3A, the subpopulations of effector/memory CD4+ T-cells (CD4^+^CD62L^−^CD44^+^) and regulatory T-cells (CD4^+^CD25^+^Foxp3^+^) in the spleen are increased, while the naive CD4+ T-cells (CD4^+^CD62L^+^CD44^−^) subpopulation is marginally reduced. Also when correcting for the increased spleen size as was found in the TAK1-Tg, increased absolute numbers of effector/memory and regulatory T-cells were found with equal amounts of naive T-cells.

Next, the B-cell subpopulations were analyzed to determine the activation status of the B-cells (see Figure 3B). Both the fraction and absolute numbers of marginal zone B-cells were elevated. Although the fraction of mature B-cells is not increased in the spleen, when correcting for the enlarged spleen size more mature B-cells are found. More importantly, the expression of MHCII^+^ on B-cells in the spleen was increased (see Figure 3B). The number 0f mature B-cells were also elevated in the spleen.

**Figure 4:**
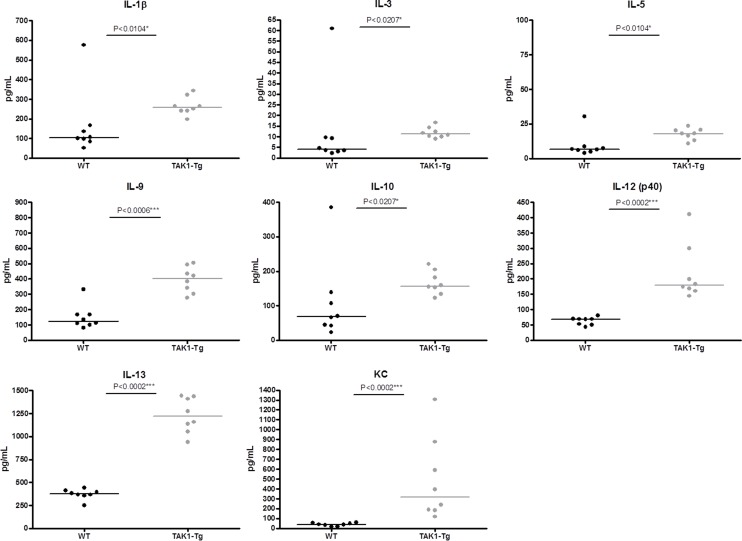
TAK1 knockdown induces circulating levels of cytokines and chemokines. Wildtype (WT) and TAK1 shRNA transgenic mice (TAK1-Tg) were treated with 0.2 mg/ml doxycycline in the drinking water for five weeks. Mice (n = 8 each group) were sacrificed and blood was harvested. Circulating levels of cytokines and chemokines were determined by Multiplex and are represented in pg/ml. Statistical significance was determined using the Mann-Whitney test (p<0.05). Data are representative of at least two independent experiments.

To study whether the elevated activation state of the immune cells translated in elevated levels of circulating cytokines, cytokine levels were quantified in the serum of TAK1-Tg and WT mice treated for five weeks with doxycycline. As shown in Figure 4, increased levels of IL-1β, IL-3, IL-5, IL-9, IL-10, IL-12(p40), IL-13 and KC were found. Taken together, our data suggest that knockdown of TAK1 protein results in an activated status of the immune system.

### TAK1 knockdown enhances acute inflammation

**Figure 5:**
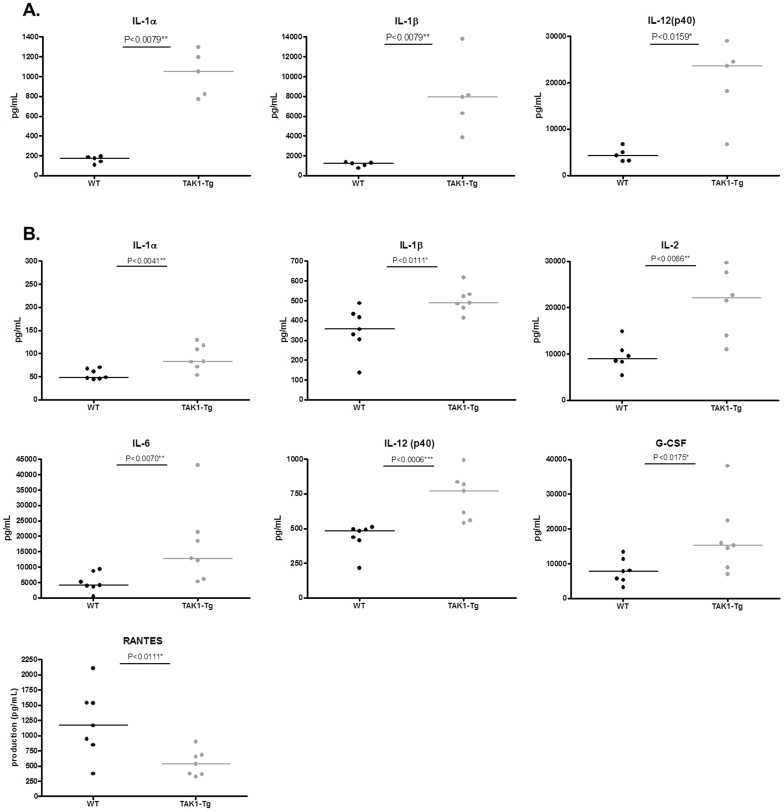
TAK1 knockdown exacerbates acute inflammation. Wildtype (WT) and TAK1 shRNA transgenic mice (TAK1-Tg) were treated with 0.2 mg/ml doxycycline in the drinking water for five weeks. Acute inflammation was induced by administration of LPS (n = 5 each group) (*A*) or anti-CD3 antibodies (n = 8 each group) (*B*) in the peritoneal cavity. After 1.5 or 3 hours, respectively mice were sacrificed and circulating levels of cytokines and chemokines were detected by Multiplex and are represented in pg/ml. Statistical significance was determined using the Mann-Whitney test (p<0.05). Data are representative of at least two independent experiments.

To study the effect of TAK1 knockdown on acute inflammation, adult TAK1-Tg mice were treated with doxycycline for five weeks and inflammation was induced by intraperitoneal injection of LPS. 1.5 hours later serum was collected to detect the secretion of cytokines/chemokines. Treatment of WT mice with doxycycline did not change serum levels of cytokines/chemokines indicating that doxycycline is not interfering with LPS responses in vivo (data not shown). TAK1-Tg mice treated with doxycycline demonstrated elevated serum levels of IL-1α, IL-1β, IL-12(p40), and RANTES (data not shown) as compared to WT mice upon stimulation with LPS (see Figure 5A). Comparing the stimulation index obtained after stimulating WT vs. TAK1-Tg with LPS, TAK1-Tg show a two-fold higher response upon LPS challenge as compared to WT mice (data not shown). In contrast, reduced levels of IL-17 (data not shown) were found.

Next, the effect of TAK1 knockdown in vivo in T-cells was studied. To establish, whether in vivo TAK1 knockdown has a functional effect in T-cells, TAK1-Tg +Dox and WT+Dox mice were challenged in vivo with anti-CD3 antibody. Circulating cytokine levels were measured in the serum of these mice, 3 hours after injection of anti-CD3 antibody. As shown in Figure 5B, TAK1 knockdown showed increased levels of circulating IL-1α, IL-1β, IL-2, IL-6, IL-12(p40), G-CSF as compared to WT +Dox mice. Also when comparing the stimulation index obtained after stimulation with anti-CD3 antibody of TAK1-Tg vs. WT mice, a two-fold higher response is observed in TAK1-Tg mice. In contrast, reduced levels of RANTES were detected in TAK1-Tg as compared to WT mice.

### TAK1 knockdown causes histopathological changes in spleen, heart, kidneys and liver

**Figure 6:**
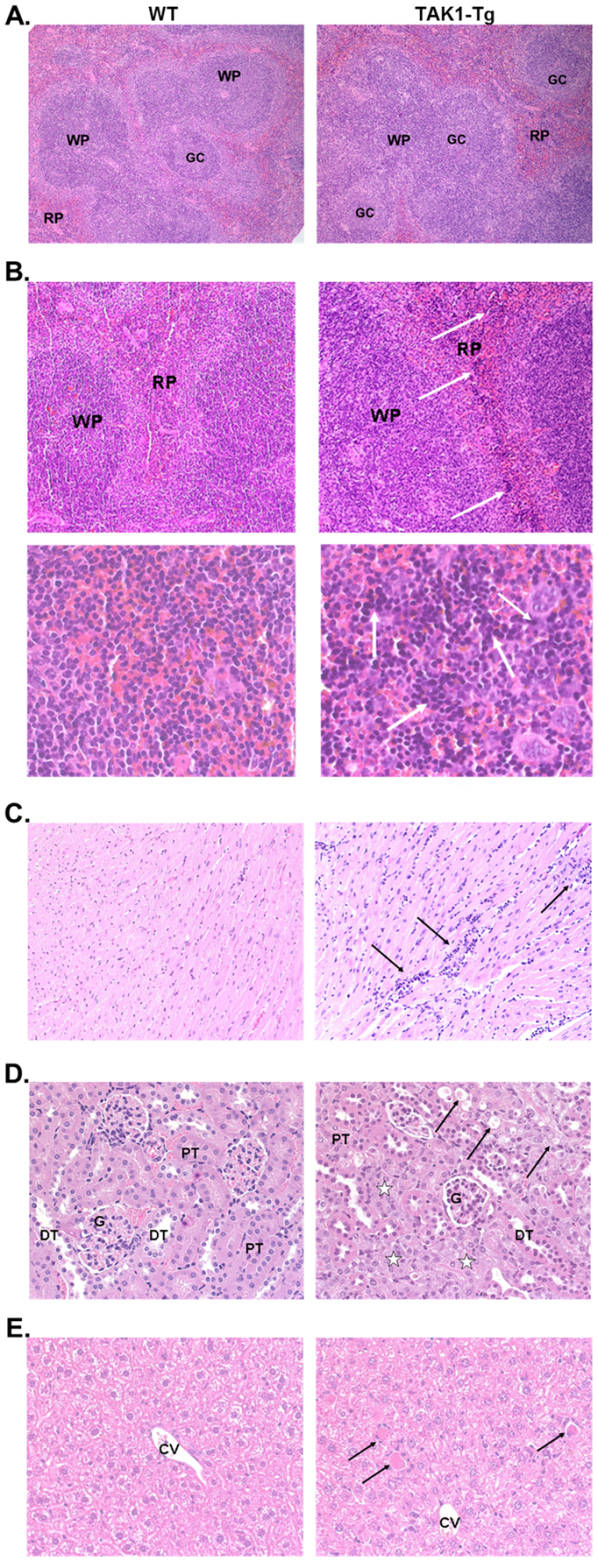
TAK1 knockdown causes histopathological changes in spleen, heart, kidneys and liver. Wild type (WT) and TAK1 shRNA transgenic mice (TAK1-Tg) were treated with 0.2 mg/ml doxycycline in the drinking water for five weeks. At autopsy, anaesthetized mice were sacrificed and organs and tissues were dissected and processed for histopathological examination (Formalin-fixed and HE-stained). *A.* Spleen (Obj. 10x). *Left,* WT: normal histological appearance of the spleen with white pulp (WP) comprising germinal centers (GC) and red pulp (RP). *Right,* TAK1-Tg: increase in cellularity of the B-cell areas and in germinal center development in the follicles. *B.* Spleen. Obj. 10x (*top*) and Obj. 40x (*bottom*). *Left*, WT: normal histological appearance of the spleen with white pulp (WP) and red pulp (RP); no extramedullary erythropoiesis. *Right,* TAK1-Tg: moderate increase in extramedullary erythropoiesis in the red pulp (arrows, *top*) showing increased numbers of erythroblasts (white arrows, *bottom*) and megakaryocytes (black arrows, *bottom*). *C.* Heart. Obj. 10x. *Left*, WT: normal histological appearance of the heart with intact muscle fibers. *Right,* TAK1-Tg: scattered slight muscle fiber degeneration and necrosis accompanied with inflammatory cells (arrows). *D.* Kidney. Obj. 40x. *Left*, WT: normal histological appearance of the kidney (cortex) with glomeruli (G), proximal (PT) and distal (DT) tubules. *Right,* TAK1-Tg: diffuse marked degeneration (arrows) and regeneration (asterisks) of the proximal tubular cells. *E.* Liver. Obj. 40x. *Left*, WT: normal histological appearance of the liver in the centrilobular region next to the central vein (CV). *Right*, TAK1-Tg: scattered several to many hepatocytes showing single cell necrosis (arrows).

To study whether knockdown of TAK-1 protein had an effect on the tissue five weeks doxycycline treated mice were sacrificed and histopathological examination of the bone marrow, heart, kidney, liver, lung, mesenteric lymph node, spleen and/or thymus was performed. In the white pulp of the spleen of all TAK1-Tg mice, an increase in cellularity of the B-cell areas and in germinal center development in the follicles was observed, suggestive of an activated B-cell phenotype (Figure 6A). Moreover, in the spleen of the TAK1-Tg mice an increase in extramedullary erythropoiesis was seen (see Figure 6B; grading was as follows: *left:* WT, no in four mice, minimal in one mouse; *right*: TAK1-Tg, slight in three mice, moderate in two mice). In the heart, three out of the five TAK1-Tg mice showed slight muscle fiber degeneration and necrosis accompanied with inflammatory cells, scattered throughout the heart (see Figure 6C). In the kidneys of all TAK1-Tg mice, diffuse marked degeneration and regeneration of the proximal tubular cells was present (see Figure 6D). In the liver of three out of five TAK1-Tg mice, several to many hepatocytes showing single cell necrosis, scattered throughout the liver, were seen. Although this lesion was not always accompanied by mononuclear cell infiltration, it has been regarded as necrosis and not as apoptosis (see Figure 6E).

No other differences in histopathology were observed between WT and TAK1-Tg mice in heart, kidney, liver, mesenteric lymph node, and/or thymus, while no differences were found in other tissues.

### TAK1 knockdown causes enhanced bone marrow proliferation and differentiation

**Figure 7:**
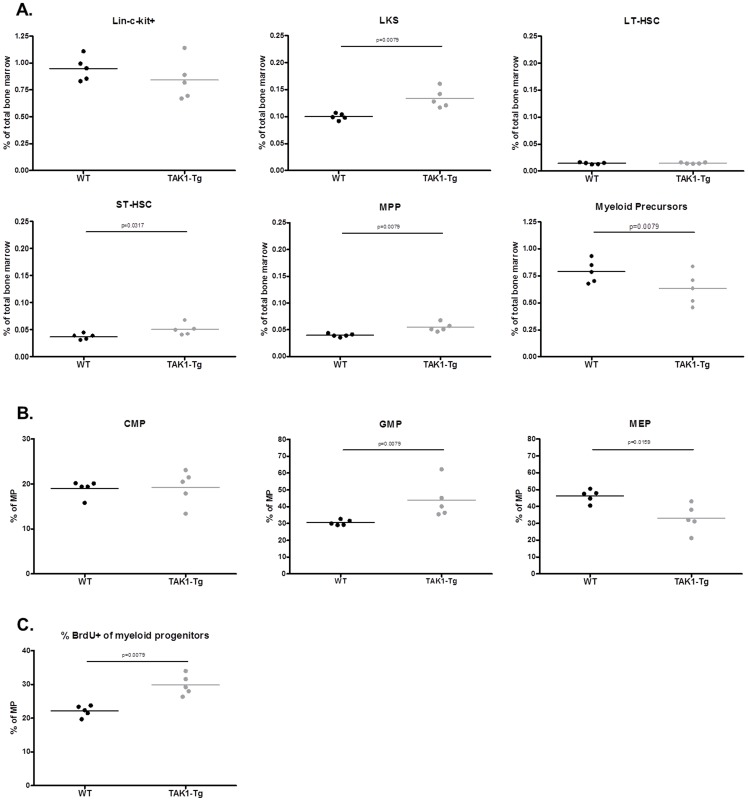
TAK1 knockdown increases proliferation and differentiation of myeloid precursors in the bone marrow. Wildtype (WT) and TAK1 shRNA transgenic mice (TAK1-Tg) were treated with 0.2 mg/ml doxycycline in the drinking water for two weeks. Mice (n = 5 each group) were sacrificed and spleens and bone marrow were harvested. (A) Lin^−^c-Kit^+^ progenitors, Lin^−^c-Kit^+^Sca-1^+^ cells (LKS population), LT-HSCs (CD34^−^Flt3^−^), ST-HSC (CD34^+^Flt3^−^), MPPs (CD34^+^Flt3^+^) and myeloid precursors (Lin^−^c-Kit^+^Sca-1^−^) were characterized by flow cytometry using the indicated cell markers. (B) Subpopulation of the myeloid precursors were characterized using the following markers: GMPs (CD34^+^CD16/32^+^), MEPs (CD34^−^CD16/32^−^) and CMPs (CD34^+^CD16/32^low^). (C). Using staining with antibodies specifically recognizing BrdU, proliferating cells in the myeloid precursors (Lin^−^c-Kit^+^Sca-1^−^) were identified. Statistical significance was determined using the two-tailed test (p<0.05).

The splenomegaly phenotype of TAK1 knockdown is already eminent after two weeks of doxycycline treatment suggesting that knockdown of TAK1 immediately affects the proliferation and/or differentiation of immune cells. To study the effect of TAK1 knockdown on the differentiation of the hematopoietic progenitor cells in the bone marrow, the subcellular composition of the bone marrow was analyzed in detail (see Figure 7). While no statistical difference in percentages of total Lin^−^c-Kit^+^ progenitors was observed between the bone marrow isolated from wild type versus two-weeks doxycycline treated TAK1-Tg mice, increased percentages of Lin^−^c-Kit^+^Sca-1^+^ cells (LKS population) were observed in the bone marrow of TAK1-Tg mice vs. WT. This increase was the result of elevated levels of ST-HSC (CD34^+^Flt3^−^) and MPPs (CD34^+^Flt3^+^), suggesting either enhanced differentiation of the most immature LT-HSCs (CD34^−^Flt3^−^) or enhanced proliferation of ST-HSC and/or MPPs (see Figure 7A). Within the myeloid precursors (Lin^−^c-Kit^+^Sca-1^−^), an increase of GMPs (CD34^+^CD16/32^+^) is observed and a decrease in MEPs (CD34^−^CD16/32^−^) in TAK1-Tg vs. WT, while no difference is seen for the percentages of CMPs (CD34^+^CD16/32^low^) (see Figure 7B). Finally, the effect of TAK1 knockdown on proliferation in the bone marrow was studied using in vivo BrdU incorporation. After 24 hours of in vivo BrdU labeling, bone marrow was analyzed for proliferating cells. As shown in Figure 7C, proliferation was enhanced in the TAK1-Tg as compared to WT mice for progenitors in the bone marrow and more specifically the proliferation of myeloid precursors was increased. No increased proliferation was seen for HSCs or MPPs (data not shown). After 24 hours increased percentages of recently proliferated neutrophils, monocytes and marginal zone B-cells were observed in spleen (data not shown). Taken together, our data suggest that systemic knock-down of TAK1-Tg knock-down results in enhanced proliferation and differentiation of precursors of the myeloid lineage in the bone marrow, at least partly explaining the splenomegaly as a result of enhanced numbers of neutrophils and monocytes.

## Discussion

To our knowledge this is the first report that describes the use of a transgenic mouse expressing a shRNA under control of an inducible promoter in the field of immunology research. Regularly, (functional) studies using conventional knock-out mice are hampered by an embryonic lethal phenotype, e.g. mice deficient in TAK1 genes die at age of E9.5 [14]. In such cases Mouse Embryonic Fibroblasts (MEFs) may be used ex-vivo to study the function of the gene of interest. Alternatively, conditional knock-out mice are generated that deplete the gene of interest in a tissue or cell-type specific manner. Although proven extremely informative, the use of these models in immunology research is in some cases restricted by the effect the genetic depletion has on the development and presence of subcellular populations, for example T-cell specific TAK1^−/−^ results in absence of peripheral T-cells. Genetic models in which depletion of the gene of interest can be induced at the desired time circumvents this problem in these cases, for example Tamoxifen inducible cre-recombinase systems [36]. In the field of kinase research additional genetic tools are available, such as the KinaseSwitch technology, which makes use of a chemical compound blocking the (mutated) kinase domain in a pharmacologically relevant setting [37]. Just like the use of pharmacological inhibitors for in vivo functional studies the use of either the tamoxifen-inducible gene knockout or the KinaseSwith technology may come with the disadvantage that the chemical compounds themselves interfere with the function of the immune system [38]. In this report we studied the use of an alternative technology, where knockdown of TAK1 is achieved in a transgenic mouse expressing a TAK1 shRNA under control of a doxycycline inducible promoter. First, we established that doxycycline itself does not affect immune responses in vivo (i.e. LPS and anti-CD3 antibody induced cytokine production; data not shown). TAK1 protein levels were reduced upon doxycycline treatment in mice, once these mice were fully matured and had developed a normal proficient immune system (data not shown). However, when taking into account that the TAK1 shRNA is expressed under control of the constitutive CAGGS promoter, it was surprising that knockdown of TAK1 mRNA and protein levels in some cases was partial and not to the same degree when comparing different tissues. In addition, in liver efficient knockdown of TAK1 mRNA did not result in equal efficient TAK1 protein knockdown. Tissue penetration of doxycycline, transcriptional activity of genomic loci in the different tissues, mRNA stability and TAK1 protein turnover time may be underlying reasons for this phenomenon. Also in other models based on the same technology [34]–[35] and MKK7 shRNA (unpublished observations)) mosaicism with respect to degree of inhibition of knockdown in different tissues was observed. In both TAK1 and MKK7 inducible shRNA transgenic models changing the dose and/or length of doxycycline treatment only had limited effect on knockdown efficiency in the different tissues. For example, increasing the dosage of doxycycline to 2 mg/ml showed a similar effect on the cellular subpopulations in the spleen and thymus (data not shown). In addition, reducing the doxycycline treatment to a two week period resulted in a similar qualitative outcome, only quantitatively less pronounced (data not shown).

Our studies demonstrate that knockdown of TAK1 results in splenomegaly. The enlarged spleens are the result of elevated numbers of neutrophils, monocytes and B-cells and/or extramedullary erythropoiesis. The systemic inflammation as detected by increased levels of circulating cytokines, chemokines and tissue infiltrating leukocytes (e.g. heart) might be the underlying cause of the accumulation of different leukocytes in the spleen. This systemic inflammation is characterized by a broad response, where Th1/Th17 promoting cytokines (IL-1β and IL-12 (p40)), Th2 signature cytokines (IL-5, IL-9, IL-13 and IL-10) and unbiased cyto-/chemokines such as KC and IL-3 were detected. In addition, data were provided suggesting this phenotype is explained by increased differentiation and proliferation of progenitor cells in the bone marrow. The other potential cause of the splenomegaly is formed by the observation of extramedullary erythropoiesis as found in the pathological examination of TAK1-Tg tissues, which is most likely due to stress-mediated erythropoiesis and linked to the systemic inflammation of the mice.

Not surprisingly, the increased immune activation status in mice upon TAK1 knockdown mice prior to immune challenges was amplified when stimulated with either LPS or anti-CD3 stimulations in vivo. Taken together our data suggest that systemic knockdown of TAK1, although not complete in every cell and tissue results in a pro-inflammatory phenotype. That TAK1 depletion results in a pro-inflammatory phenotype was reported before in models where TAK1 was specifically depleted in skin, intestine or liver. Skin-specific depletion of TAK1 causes a severe skin inflammation 6–8 days postnatal [16]–[17], whereas in the intestines TAK1 depletion resulted in intestinal inflammation [21]–[23]. Finally, liver depletion of TAK1 resulted in apoptotic cell death of hepatic cells, resulting in liver inflammation [27], [29]. Although our studies confirmed a pro-inflammatory phenotype, this phenotype did not translate in pathological changes in the skin or intestine. The absence in our model of efficient TAK1 knockdown in the skin (Figure 1) may explain this difference. Although not reported here, our studies do not completely rule out the absence of an effect of TAK1 knockdown in intestine. Preliminary data are available that suggest that long-term treatment (3 months) of TAK1-Tg mice vs. WT mice causes wasting syndrome in 5/10 TAK1-Tg mice (characterized by >15% weight loss) as compared to no weight loss in WT mice (unpublished observations).

In contrast with other models, our data could not confirm a function of TAK1 protein in thymocyte development, peripheral T-cells function or Toll-Like receptor signaling (LPS). Studies using TAK1^−/−^ MEFs and B-cell specific depletion of TAK1 suggested that TAK1 is involved in pro-inflammatory signal transduction upon Toll-Like and B-cell Receptor activation [14], [26]. In a setting where TAK1 is depleted in T-cells, TAK1 appeared to be indispensable for the development of thymocytes and peripheral T-cells resulting in the development of colitis, probably due to the lack of central Tregs [18]–[19]. More detailed studies indicated that TAK1 is required for T-cell receptor and IL-7 induced signaling in mature thymocytes, whereas in effector T-cells TAK1 is required for IL-2, IL-7 and IL-15 induced signal-transduction and not for T-cell receptor induced NF-κB and cytokine production [20]. In macrophages specific knockdown of TAK1 resulted in a decrease of Th1 and Th17 cells and thereby inhibition of the development of experimental arthritis [30]. One explanation for the apparent controversy may be formed by the partial and/or cell-specific knockdown obtained upon doxycycline treatment as compared to the complete absence of TAK1 in conditional knockouts. Alternatively, the inflamed status of the mouse may preclude and overrule an effect of TAK1 knockdown in specific signal transductions such as T-cell receptor or Toll-Like receptor 4 triggering. Increased circulating levels of IL-1β, IL-12(p40), IL-13 and KC either alone or in combination with increased numbers of neutrophils, monocytes, effector/memory T-cells and marginal zone B-cells may overrule a potential anti-inflammatory effect of TAK1 knockdown in T-cell or Toll-like receptor signaling.

Recently, data were reported that are confirming our observations. Using a mouse models in which TAK1 was ablated in myeloid cells, splenomegaly, extramedullar hematopoiesis, enlarged lymph nodes, massive myeloid hyperplasia, due to hyperproliferation and increased numbers of activated T-cells were observed [39]. In accordance, enhanced responses upon LPS challenge were observed. In contrast to our observations, for the myeloid-specific TAK1 knockout the systemic inflammation (pathological observations in heart, kidney and liver and elevated levels of circulating cytokines in serum) that was observed in the TAK1-Tg, without any immunological challenge was not observed as well as the B-cell (hyperproliferation, activation and germinal center formation) and CD4+ phenotype. In addition, we demonstrated that also upon T-cell challenge, elevated responses were detected upon TAK1 knockdown. Taken together, it appears that the TAK1-Tg model demonstrates a more pronounced, but similar pro-inflammatory phenotype than the *Map3k7* ^ΔM/ΔM^ mouse model. In both model the increased proliferation of the myeloid precursors in the bone marrow is suggested to form the underlying cause of this phenotype. The knockdown in other cell-types than myeloid cells obtained in the TAK1-Tg (e.g. strong knockdown observed in splenic B- and T-cells) might explain the enhanced or additional phenotype: increased circulating cytokines/chemokines, tissue inflammation, degeneration of kidney tubuli and enhanced numbers and activation of B-cell in spleen and blood.

Originally, TAK1 kinase was identified as TGF-β activated kinase 1 [6]. When comparing the effect of genetic models of TGF-β1 and TGF-β receptor chains with the TAK1 shRNA knockdown similar phenotypes are observed. Mice genetically depleted for TGF-β1 succumb to a wasting syndrome and systemic inflammation, which is characterized by increased levels of cytokines (e.g. IL-1β), increased numbers of circulating neutrophils and monocytes and tissue infiltrating leukocytes (particularly heart, lung and stomach) [40]–[42]. In addition, autoantibodies suggestive of SLE-like and Sjögren's Syndrom-like phenotype were observed in these mice [43]–[44]. In models where a dominant negative form of the TGF-β receptor Type II (dnTGFβRII) was overexpressed in T-cells similar phenotypes were observed: wasting, mononuclear cell infiltration in multiple organs and autoantibodies. In addition, enlarged spleens and increased numbers of effector/memory CD4+ T-cells [45]. Subsequent studies using retroviral overexpression dnTGFβRII in the bone marrow demonstrated expansion of the splenic myeloid cells and systemic inflammation, eventually resulting in dramatic weight loss [46]. Letterio and coworkers elegantly demonstrated that the accelerated myelopoiesis in the bone marrow of TGF-β1^−/−^ is occurring in the absence of systemic inflammation and exposure to cytokines [47]. Our data demonstrate a similar effect of TAK1 knockdown in the bone marrow, where enhanced proliferation and differentiation is resulting in a dramatic increase of neutrophils and monocytes, suggesting that the phenotype of TAK1 knockdown is (at least partially) mediated via its function in downstream signaling of TGF-β. This is also supported by our observations in the kidney, where tubuli degeneration was found, a phenotype that was also described for TGF-β2 null mice [48].

Finally, we undertook our studies to reveal the function of TAK1 and to answer whether systemic inhibition of TAK1 has a pro- or anti-inflammatory effect. Extensive literature data have demonstrated that TAK1 kinase is an important node in pro-inflammatory signal transduction upon the activation of many receptors (T-cell, B-cell, NOD2, Toll-Like and cytokine receptors) [8,−10]. In addition, TAK1 was recently linked to human pathology, i.e. rheumatoid arthritis [31], [33]. Especially, with disappointing results of limited efficacy of p38 inhibitors, a downstream kinase of TAK1 in Phase 2 clinical trials for rheumatoid arthritis discussions have suggested to go upstream to obtain efficacy [33]. In fact, molecular studies indicate that p38 inhibitors via a feedback control mechanism (TAB1 protein) results in enhanced activity of TAK1, thereby activating other downstream kinases of TAK1, such as JNK and IκB kinase [49]. However, our data and the data provided by Alagbala Ajibade and coworkers in fact support the notion that TAK1 inhibition results in a pro-inflammatory environment [39]. Alagbala Ajibade and coworkers elegantly demonstrated that depletion of TAK1 results in enhanced p38 phosphorylation and NF-κB activation, thereby resulting in enhanced immune activation upon LPS challenge. Taken together with the observations that tissue-specific depletion of TAK1 results in enhanced inflammation, pharmaceutical inhibition of TAK1 will most likely enhance inflammation and as such is invalidating the hypothesis that TAK1 inhibition might form an alternative to p38 inhibitors for treatment of autoimmune diseases. We suggest, based on the similarities between our observations and TGF-β genetic models, that TAK1 has a more prominent function in TGF-β signaling than was thus far appreciated.
